# Fetal growth restriction and the risk of perinatal mortality–case studies from the multicentre PORTO study

**DOI:** 10.1186/1471-2393-14-63

**Published:** 2014-02-11

**Authors:** Julia Unterscheider, Keelin O’Donoghue, Sean Daly, Michael P Geary, Mairead M Kennelly, Fionnuala M McAuliffe, Alyson Hunter, John J Morrison, Gerard Burke, Patrick Dicker, Elizabeth C Tully, Fergal D Malone

**Affiliations:** 1 Obstetrics & Gynaecology, Royal College of Surgeons in Ireland, Dublin, Ireland; 2 Obstetrics & Gynaecology, University College Cork, Cork University Maternity Hospital, Cork, Ireland; 3 Obstetrics & Gynaecology, Coombe Women and Infants University Hospital, Dublin, Ireland; 4 Obstetrics & Gynaecology, Rotunda Hospital, Dublin, Ireland; 5 UCD Centre for Human Reproduction, Coombe Women and Infants University Hospital, Dublin, Ireland; 6 Obstetrics & Gynaecology, UCD School of Medicine and Medical Science, National Maternity Hospital, Dublin, Ireland; 7 Obstetrics & Gynaecology, Royal Jubilee Maternity Hospital, Belfast, Ireland; 8 Obstetrics & Gynaecology, National University of Ireland, Galway, Ireland; 9 Obstetrics & Gynaecology, Graduate Entry Medical School, University of Limerick, Limerick, Ireland; 10 Epidemiology & Public Health, Royal College of Surgeons in Ireland, Dublin, Ireland

**Keywords:** Perinatal mortality, Antepartum stillbirth, Neonatal death, Intrauterine growth restriction, PORTO Study

## Abstract

**Background:**

Intrauterine growth restriction (IUGR) is the single largest contributing factor to perinatal mortality in non-anomalous fetuses. Advances in antenatal and neonatal critical care have resulted in a reduction in neonatal deaths over the past decades, while stillbirth rates have remained unchanged. Antenatal detection rates of fetal growth failure are low, and these pregnancies carry a high risk of perinatal death.

**Methods:**

The Prospective Observational Trial to Optimize Paediatric Health in IUGR (PORTO) Study recruited 1,200 ultrasound-dated singleton IUGR pregnancies, defined as EFW <10^th^ centile, between 24^+0^ and 36^+6^ weeks gestation. All recruited fetuses underwent serial sonographic assessment of fetal weight and multi-vessel Doppler studies until birth. Perinatal outcomes were recorded for all pregnancies. Case records of the perinatal deaths from this prospectively recruited IUGR cohort were reviewed, their pregnancy details and outcome were analysed descriptively and compared to the entire cohort.

**Results:**

Of 1,116 non-anomalous singleton infants with EFW <10^th^ centile, 6 resulted in perinatal deaths including 3 stillbirths and 3 early neonatal deaths. Perinatal deaths occurred between 24^+6^ and 35^+0^ weeks gestation corresponding to birthweights ranging from 460 to 2260 grams. Perinatal deaths occurred more commonly in pregnancies with severe growth restriction (EFW <3^rd^ centile) and associated abnormal Doppler findings resulting in earlier gestational ages at delivery and lower birthweights. All of the described pregnancies were complicated by either significant maternal comorbidities, e.g. hypertension, systemic lupus erythematosus (SLE) or diabetes, or poor obstetric histories, e.g. prior perinatal death, mid-trimester or recurrent pregnancy loss. Five of the 6 mortalities occurred in women of non-Irish ethnic backgrounds. All perinatal deaths showed abnormalities on placental histopathological evaluation.

**Conclusions:**

The PNMR in this cohort of prenatally identified IUGR cases was 5.4/1,000 and compares favourably to the overall national rate of 4.1/1,000 births, which can be attributed to increased surveillance and timely delivery. Despite antenatal recognition of IUGR and associated maternal risk factors, not all perinatal deaths can be prevented.

## Background

Suboptimal intrauterine growth affects up to 10% of pregnancies and confers an increased risk of perinatal morbidity and mortality. The perinatal outcome of IUGR fetuses is largely dependent on the severity of growth restriction with those below the 3^rd^ centile and/ or abnormal umbilical artery Doppler measurements at greatest risk of adverse outcome [1]. Other important prenatal determinants of perinatal outcome are gestational age at delivery and birthweight, with best prospects of morbidity-free survival to hospital discharge at weights over 800 grams and gestational ages over 29 weeks [2].

Advances in obstetrical and critical neonatal care are reflected in a substantial decrease in the overall perinatal mortality rate (PNMR) in high income countries. While this effect is mainly seen in a reduction of early neonatal deaths, stillbirth rates have remained largely unchanged over the past years [3,4]. In addition to congenital abnormalities, more recent reports have identified fetal growth restriction as one of the main contributors to perinatal mortality [5].

Ireland consistently reports highest birth rates among European countries with 16.8 births per 1,000 population (compared to 13.0/1,000 in the UK and 8.3/1,000 in Germany) [6]. In 2011, there were 74,265 births and 456 perinatal deaths in Ireland [7]. Stillbirths and early neonatal deaths accounted for 70% (n = 318) and 30% (n = 138) of perinatal deaths respectively, corresponding to a PNMR of 6.1/1,000 births. Of the 456 perinatal deaths, 155 (34%) were attributed to congenital structural or genetic abnormalities (corrected PNMR 4.1/1,000). Over 50% of infants affected by perinatal deaths in 2011 were identified as having birthweights below the 10^th^ customised centile and only 30% of those were suspected antenatally [7].

Current antenatal detection rates of IUGR are reported at 25 to 36% [8,9]. Therefore, a preventative strategy to reduce stillbirths is to improve the antenatal detection of fetal growth failure. The risk of stillbirth in pregnancies with prenatally identified IUGR is 1% (9.7/1,000 births). Pregnancies with unrecognized IUGR carry an over 8-fold increased risk of stillbirth (SB) when compared to pregnancies without IUGR (19.8 versus 2.4/1,000 births) [5]. Whenever IUGR is diagnosed prenatally, increased surveillance and timely delivery aims to improve perinatal outcome in IUGR, balancing the risk of antepartum stillbirth by remaining in utero and iatrogenic prematurity potentially causing significant morbidity or neonatal death by too early intervention.

This paper describes the 6 perinatal mortality cases which occurred in the setting of a multicentre prospective study of 1,200 pregnancies with prenatally identified IUGR defined as EFW below the 10^th^ centile [1,10].

## Methods

The Prospective Observational Trial to Optimise Paediatric Health in IUGR (PORTO) is a national prospective study conducted at seven largest maternity units in Ireland. Intrauterine growth restriction (IUGR) was defined as EFW below the 10^th^ centile based on sonographic measurements of the fetal head circumference (HC), bi-parietal diameter (BPD), abdominal circumference (AC) and femur length (FL) according to population-based growth standards (Hadlock-4) [11]. Between January 2010 and June 2012, the PORTO Study recruited 1,200 ultrasound-dated singleton pregnancies between 24^+0^ and 36^+6^ weeks’ and an EFW equal or greater than 500 grams. Fetuses with congenital structural or chromosomal abnormalities were excluded from the final analysis. Ethical approval was obtained at each participating site, and a written informed consent was given by all study participants.

Women with IUGR pregnancies underwent serial sonographic evaluation of fetal weight at 2-weekly intervals until birth including evaluation of amniotic fluid volume and multi-vessel Doppler assessment. Details on Doppler assessment and reference ranges are described in a previous publication [10]. In cases of absent (AEDF) or reversed end-diastolic (REDF) in the UA, the patient was admitted to hospital and daily electronic fetal heart rate (FHR) monitoring with computerised analysis of short term variation according to Dawes/Redman criteria (Sonicaid Team Fetal Monitor with FHR analysis, MDI Medicare, Ireland) was carried out. Corticosteroids to promote fetal lung maturation and reduce perinatal morbidity were administered as a single course between 24 and 36 weeks’ gestation if delivery was likely within one week. Given that the PORTO Study was observational and descriptive in nature, there were no pre-specified management or delivery criteria and all decisions were made by the lead clinician managing each case reflecting real world clinical practice. All study participants received the same surveillance (2-weekly sonogram or more frequently as deemed necessary) with the aim to describe differences in outcome. There was however general agreement among clinicians to deliver AEDF cases no later than 34 weeks’ gestation.

The perinatal mortality rate (PNMR) was calculated by the number of stillbirths and neonatal deaths per 1,000 live births and stillbirths (all births were ≥24 weeks gestation and weighed ≥500 grams). The PNMR was corrected for congenital abnormalities.

All cases were prospectively recruited and medical records of all mortalities were reviewed retrospectively for this descriptive analysis. Comparisons were made to the entire cohort, and demographic and pregnancy course and outcome data were recorded along with a review of placental histopathology.

Placental abnormalities were categorised according to Redline criteria [12] into villous developmental abnormalities (distal villous dysmaturity/immaturity, abnormal placental shape/position, chorangiosis), maternal vascular pathology, fetal vascular pathology and inflammatory lesions (acute chorioamnionitis, chronic villitis of unknown aetiology). Maternal vascular injuries include infarction, retroplacental haemorrhage and increased perivillous fibrinoid deposition (maternal floor infarction). Fetal vascular injuries include true cord knots, cord hypercoiling, abnormal cord insertion, single umbilical artery and fetal thrombotic vasculopathy.

**Figure 1:**
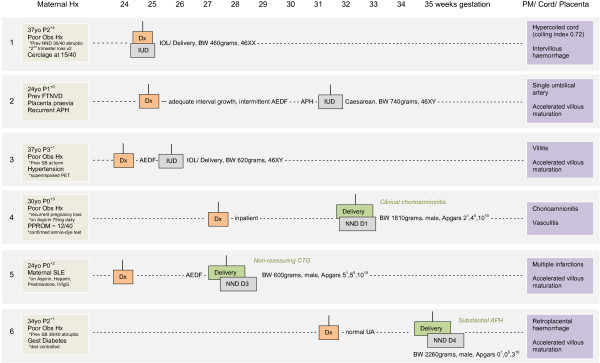
**Case study of non-anomalous singleton PORTO mortalities (n = 6).** SB stillbirth; NND neonatal death, D day of life; Dx diagnosis; IUD intrauterine death; AEDF absent end-diastolic flow; UA umbilical artery; Obs Hx obstetric history; PET pre-eclampsia; Gest gestational; Prev previous; APH antepartum haemorrhage; BW birthweight; IOL induction of labour; CTG cardiotocograph.

Due to the small sample size, formal statistical comparisons and modelling (such as logistic regression) are underpowered. Therefore, statistically non-significant findings have the potential to be misleading. Furthermore, due to the biases associated with retrospective selection of controls and the potential for overmatching with a small case-set, the mortalities are presented as a case-series rather than as a case-control study. Consequently, the cases have been described individually (as shown in Figure 1), where each pregnancy/mortality case has its own context.

## Results

There were 6 mortalities including 3 stillbirths (SB) and 3 early neonatal deaths (NND) within the entire cohort of 1,116 non-anomalous singleton pregnancies completing the study protocol. This corresponds to a PNMR of 5.4/1,000. The maternal demographics, baseline characteristics and sonographic findings are described in Table 1. Not surprisingly, perinatal deaths occurred more commonly in pregnancies with severe growth restriction (EFW <3^rd^ centile) and associated abnormal Doppler findings resulting in earlier gestational ages at delivery and lower birthweights.

**Table 1:** Maternal demographics, baseline characteristics and sonographic findings

	**Perinatal mortalities (n = 6)**	**Alive at hospital discharge (n = 1,110)**
Maternal age	31.5 ± 6.3	29.9 ± 6.0
Advanced maternal age >35 years	2 (33%)	225 (20%)
Nulliparity	2 (33%)	298 (45%)
Smoker	0	261 (24%)
BMI >30 kg/m^2^	3 (50%)	122 (11%)
Hypertension/Pre-eclampsia	1 (17%)	148 (13%)
Placental abruption	2 (33%)	14 (1%)
Assisted reproductive techniques	0	16 (1%)
Maternal diabetes	1 (17%)	17 (2%)
GA at enrolment	26.1 ± 2.7	30.1 ± 3.9
GA at delivery	29.4 ± 3.8	37.8 ± 2.9
Last scan EFW <3^rd^ centile	5 (83%)	421 (38%)
Last scan EFW <10^th^ centile	6 (100%)	834 (75%)
Last scan EFW <10^th^ customised centile	6 (100%)	794 (72%)
Birthweight <3^rd^ centile	4 (67%)	507 (46%)
Abnormal UA Doppler (PI >95^th^, AEDF)	4 (67%)	508 (46%)
AEDF in the UA	4 (68%)	72 (6%)

*PI*, pulsatility index; *UA*, umbilical artery; *AEDF*, absent end-diastolic flow; *EFW*, estimated fetal weight; *GA*, gestational age.

Stillbirths occurred at gestational ages between 24^+6^ and 31^+3^ weeks with birthweights ranging from 460 to 740 grams (mean 607 grams). All stillbirths had estimated weights below the 3^rd^ centile and AEDF in the umbilical artery, and they occurred during inpatient care. The median time from study recruitment to diagnosis of intrauterine demise was 12 days (range 0-45 days). One woman had placenta praevia associated with recurrent antepartum haemorrhages and one woman had severe pre-eclampsia on the background of essential hypertension. Two women had previous pregnancies that resulted in perinatal deaths (one NND due to abruption at 36 weeks, one with unexplained SB at term), and all women with stillbirths were non-Irish nationals.

All cases of neonatal deaths were delivered by emergency caesarean section between 27^+4^ and 35^+0^ weeks gestation corresponding to birthweights between 600 and 2260 grams (mean 1147 grams). The median time of antenatal surveillance was 25 days (range 24-34 days). As outlined in Figure 1, delivery #4 was for clinical chorioamnionitis due to prolonged preterm rupture of membranes from 12 weeks gestation. The neonate died on day 1 of life due to pulmonary hypoplasia. A prolonged bradycardia on antenatal fetal heart rate monitoring prompted delivery of a severely growth restricted infant <3^rd^ centile and umbilical artery AEDF in case #5. The mother received inpatient care for active SLE and the neonate died on day 3 of life. Neonatal death #6 resulted from a recurrent placental abruption at 35 weeks gestation one week prior to her planned delivery; in this case neonatal intensive care was withdrawn after 4 days due to grade 3 hypoxic ischemic encephalopathy (HIE) and multi-organ failure.

Figure 1 summarises the obstetric history and maternal risk factors and outlines the pregnancy course in detail, including information on delivery indication and outcome. The table also shows information derived from post-mortem examination, cord and placental histopathological evaluation. All of the described pregnancies were complicated by either significant maternal comorbidities, in particular hypertension, SLE or diabetes, or poor obstetric histories. Maternal histories were significant for previous IUGR, recurrent or late pregnancy loss, NND or SB. Two thirds of patients (n = 4) had a poor obstetric history including 3 women with term mortalities in prior pregnancies (2 placental abruptions and 1 unexplained stillbirth at term) and 1 woman with a history of recurrent first trimester miscarriages. Four of the 6 mortalities occurred in women of non-Irish ethnic backgrounds. All cases showed placental or cord pathologies including maternal and fetal vascular injuries, placental developmental abnormalities or inflammatory lesions.

The majority of perinatal deaths (n = 5) were associated with an EFW < 3^rd^ centile and abnormal UA Doppler velocimetries in the umbilical artery. The 2 perinatal deaths with normal UA Doppler were attributed to pulmonary hypoplasia and histologically confirmed chorioamnionitis due to prolonged preterm rupture of the membranes which and to a recurrent placental abruption at 35 weeks gestation.

## Discussion

The striking and common feature of all perinatal death cases in our study is that they more commonly occurred in non-Irish national women attending for antenatal care with poor obstetric histories and coexistent maternal co-morbidities. These findings are in agreement with other reports which found that ethnic minorities are an overrepresented group among mothers who experience perinatal deaths [7,13]. In particular, women with a history of adverse pregnancy outcomes, such as recurrent pregnancy loss, stillbirth or perinatal deaths, are at higher risk of recurrent adverse outcomes with fewer than 25% pregnancies resulting in surviving infants [14].

Perinatal deaths occurred more commonly among infants with severe growth restriction and associated abnormal umbilical artery Doppler values which highlights the fact that fetuses with EFW <3^rd^ centile and/or abnormal UA Doppler are a greater risk of poor perinatal outcome therefore making this a more appropriate cut-off for the definition of pathological growth restriction [1].

A detailed evaluation of the cord and placenta is useful in determining the underlying causes which have led to an IUGR diagnosis. Histopathological examination was carried out for all perinatal deaths in our study and interestingly showed abnormal findings in 100% of cases including maternal and fetal vascular injuries, placental developmental abnormalities or inflammatory lesions. Unfortunately, there are no specific antenatal screening or preventative strategies for cord and placental abnormalities. Together with maternal factors, this information can however be utilised when caring for women in a subsequent pregnancies.

The PNMR in this IUGR cohort compares favourably to the national corrected PNMR of 4.1/1,000. The relatively low PNMR in this high risk population of IUGR fetuses can be explained by (i) the intensive prenatal surveillance within a research setting attributed to the Hawthorne effect, which has been described as a confounding factor for performance in clinical observational studies and (ii) the exclusion of pregnancies at most severe spectrum of disease (i.e. EFW <500 grams at or over 24 weeks gestation). Despite the antenatal recognition of maternal risk factors and diagnosis of IUGR with initiation of increased surveillance and inpatient management, not all perinatal deaths in our study were prevented. Some of these deaths occurred during inpatient care or were due to unforeseen acute complications such as placental abruption, antepartum haemorrhage in the presence of placenta praevia or acute chorioamnionitis. However the increased surveillance certainly benefited the majority of women and babies enrolled in PORTO reflected in the low PNMR in this high risk cohort which was comparable to the overall national rate.

**Figure 2:**
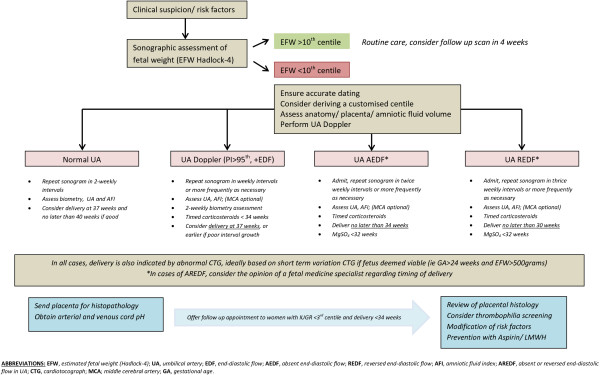
Fetal growth restriction–management algorithm.

A limitation of our study concerns the inability to comment on antenatal detection rates of IUGR in Irish maternity units as data on this was not recorded. Prenatal recognition of IUGR remains the main challenge in daily obstetric practice. Current detection rates of IUGR in the antenatal setting are at best 36% and future research needs to urgently focus on how we can improve these detection rates. Clinical examination, abdominal palpation and fundal height measurement have limited accuracy in identifying IUGR prenatally and serial ultrasound scanning from 26 to 28 weeks of gestation has been proposed in patients with risk factors [15]. A care model whereby every pregnant patient receives an ultrasound scan at least 4 weekly intervals would improve identification of growth failure based on population and customised growth standards. Given that IUGR can also occur in infants born with birthweights above the 10^th^ centile cut-off, this approach would also allow to comment on growth trajectories which have been identified as an important factor in the prediction of morbidity and mortality outcomes [16]. The relevance to clinical practice in reducing perinatal morbidity and mortality could be the subject of future research studies comparing various models of antenatal care. This will impact on resource issues, increase obstetric intervention but no doubt will have an impact on the antenatal detection and reduction in perinatal deaths. At the present time, we propose a management algorithm outlined in Figure 2[17].

## Conclusions

Fetal growth restriction is the single most important contributor to perinatal mortality in non-anomalous fetuses [5]. Significant research efforts have gone into improving the diagnosis and definition of IUGR, surveillance and antenatal management, however uncertainties regarding the optimal timing of delivery in IUGR persist.

Vigilance towards antenatal risk factors for poor pregnancy outcome is important for the optimal management of IUGR pregnancies. The focus of future research needs to urgently address how we can improve the antenatal detection of IUGR. Prenatal recognition of IUGR dramatically improves pregnancy outcomes allowing for close monitoring and timely delivery. Nevertheless, even if recognised, not all perinatal deaths can be prevented.

## Abbreviations

IUGR: Intrauterine growth restriction; PORTO: Prospective Observational Trial to Optimize Paediatric Health in IUGR; SB: Stillbirth; IUD: Intrauterine death; UtA: Uterine artery; UA: Umbilical artery; NND: Neonatal death; APH: Antepartum haemorrhage; BW: Birthweight; Dx: Diagnosis; PET: Pre-eclampsia; PPROM: Prolonged preterm rupture of membranes; AEDF: Absent end-diastolic in the UA; REDF: Reversed end-diastolic flow in the UA; CTG: Computerised short term variation cardiotocograph; HIE: Hypoxic ischaemic encephalopathy; PNMR: Perinatal mortality rate.

## Competing interests

The authors declare that they have no competing interests. The study was funded by the Health Research Board (HRB) and Friends of the Rotunda.

## Authors’ contributions

All authors have made substantial contributions to conception, design and data acquisition of the PORTO study. KOD had the idea for this particular analysis. JU drafted the manuscript. All co-authors revised it critically for important intellectual content. PD performed the statistical analysis. All authors read and approved the final manuscript.

## Authors’ information

The PORTO Study was conducted by the Perinatal Ireland Research Consortium, a nationwide collaborative research network comprising of the seven largest academic obstetric centres in Ireland. Those include the Rotunda Hospital Dublin, the Coombe Women’s and Infants University Hospital Dublin, the National Maternity Hospital Dublin, the Cork University Maternity Hospital, the Royal Jubilee Maternity Hospital Belfast, the Galway University Hospital and the Mid-Western Regional Maternity Hospital Limerick. FDM is the Chair of Perinatal Ireland. JU is the lead researcher of the PORTO study and clinical PhD student under the supervision of KOD and FDM.

## Pre-publication history

The pre-publication history for this paper can be accessed here:

http://www.biomedcentral.com/1471-2393/14/63/prepub
